# Safety in magnetic resonance units: an update

**DOI:** 10.1111/j.1365-2044.2010.06377.x

**Published:** 2010-07

**Authors:** PA Farling, PA Flynn, G Darwent, J De Wilde, D Grainger, S King, ME McBrien, DK Menon, JP Ridgway, M Sury, J Thornton, SR Wilson, Association of Anaesthetists of Great Britain and Ireland

## Abstract

The number of anaesthetists who are involved in magnetic resonance (MR) units is increasing. Magnetic resonance systems are becoming more powerful and interventional procedures are now possible. This paper updates information relating to safety terminology, occupational exposure, reactions to gadolinium-based contrast agents and the risk of nephrogenic systemic fibrosis. Magnetic resonance examinations of patients with pacemakers are still generally contra-indicated but have been carried out in specialist centres under strictly controlled conditions. As availability of MR increases, so the education of anaesthetists, who are occasionally required to provide a service, must be considered.

Anaesthesia in MR units was first described in the 1980s. Guidelines on the provision of anaesthetic services in MR units were published by the Association of Anaesthetists of Great Britain and Ireland (AAGBI) in 2002 [1]. Since then, the number of hospitals with MR units, and hence the number of patients requiring anaesthesia for MR, has increased. While the issues relating to setting up anaesthetic services in MR have not changed, there have been a number of developments that warrant this update:

1. Safety terminology and guidelines have changed.
2. MR systems utilise higher magnetic-field strengths and more open designs are available.
3. Interventional and intra-operative MR are now routine in some centres.
4. Mobile MR scanners are increasingly used to reduce waiting lists.
5. Although still generally contra-indicated, some patients with pacemakers have been scanned under strictly controlled conditions in specialist centres.
6. ‘MR safe’ medical implants are now being produced.
7. New equipment is now available for use in MR.
8. Out-of-hours availability of MR investigations has increased.
9. Reports of allergic reactions to MR contrast media have increased.
10. Gadolinium based contrast agents (Gd-CAs) are associated with a varying degree of risk of nephrogenic systemic fibrosis in patients with impaired renal function.

## Safety guidelines and legislation

In 2007 the Medicines and Healthcare products Regulatory Agency (MHRA) updated safety guidance as a Device Bulletin [2]. Three terms are now to be used as standard in an attempt to remove any ambiguity caused by the old *MR compatible* system. These terms are *MR conditional*, *MR safe* and *MR unsafe*. MR conditional refers to an item that has been demonstrated to pose no known hazards in a specified MR environment with specified conditions of use. Many items in the MR environment will now be marked as MR conditional, and the conditions under which they can be safely used must accompany the device. This change of terminology has come about because of reports of injuries and problems with MR compatible equipment [3]. Conditions that define the specified MR environment include main magnetic field strength, spatial magnetic field gradient, dB/dt (time rate of change of the magnetic field), radio frequency (RF) field strength, and specific absorption rate. Additional conditions, including specific configurations of the item of equipment, may be required.

Equipment is designated as MR safe if it presents no safety hazard to patients or personnel when it is taken into the MR examination room, provided that instructions concerning its use are correctly followed. This does not, however, guarantee that it will function normally and not interfere with the correct operation of the MR imaging equipment, with degradation of image quality.

New equipment, such as infusion pumps [4], warming mattresses and temperature probes are now available. It is important to understand the manufacturers’ instructions of all equipment that is brought into the vicinity of the MR scanner.

It should be recognised that the supervising MR radiographer is responsible operationally for MR safety within the controlled area and that anaesthetic staff should defer to him/her in relation to MR safety matters, in particular control of access of staff and equipment into the controlled area. Where staff are given access codes or swipe-card access to the controlled area, they should not be shared with others, nor should they provide access to others unless specifically authorised to do so.

### Inspired oxygen concentration

The use of 100% O_2_ during anaesthesia should be reported to the reporting radiologist as this can produce an artefact in the form of an abnormally high signal in cerebrospinal fluid (CSF) spaces in the T2 weighted fluid attenuated inversion recovery (FLAIR) sequence.

### Acoustic noise

The time-varying magnetic field gradients produce audible noise within the magnet interior. Since the guidelines were published by the AAGBI, the Control of Noise at Work Regulations have been updated [5]. This document introduced lower exposure limit values and action values in the working environment. When the noise level exceeds 80 dB (A), it is recommended that staff and others remaining in the scanning room should wear ear protection.

Other documents have been published by the Health Protection Agency relating to patient exposure guidance [6] and static field guidance [7]. The website of the British Association of MR Radiographers (BAMRR) remains an excellent resource for safety issues and provides links to many useful safety sites [8].

## New MR systems

At the end of 2006, it was estimated that there were approximately 500 fixed MR scanners involved in human imaging, installed at some 350 sites across the UK [6]. The SI unit of magnetic field strength or magnetic flux density is the Tesla (T) and initially, most clinical MR systems were 0.5, 1.0 or 1.5 T. In 1992 there were two MR units in Northern Ireland. Today there are 16, of which, one is a 3-T system. Other regions will have experienced a similar expansion but, since the withdrawal of funding from MagNET, up-to-date information for the UK is difficult to obtain.

Magnets operating at 3 T appeared in the early 1990s and by 2007 it was estimated that 35 units had installed 3-T systems [5]. The benefits of the higher field strength systems include improved image quality and higher spatial resolution. While it is claimed that 3-T scans are quicker, more efficient and require less Gd-CAs, practically these statements are debatable. It is the responsibility of the equipment manufacturers to indicate the field strength at which their equipment is MR safe or MR conditional. It should not be automatically assumed that equipment that is MR conditional at 1.5 T remains MR conditional at 3 T. A smaller number of ultra-high field MR systems are in use in research institutions world wide and these produce static fields in the range 4.7–9.4 T [6]. Anaesthetists may wish to be aware of the potential implications of replacing a 1.5-T system by a 3-T system. In a magnetic field strength survey of a 1.5-T system all spot measurements taken at 1 m above the floor level were found to be below the 0.5-mT safety limit. A similar survey for a 3-T system indicated that there were areas outside the magnet room where levels exceeded the safety limit. Barriers and warning notices, which indicate the risk of pacemaker malfunction, should be in place to prevent inadvertent public access.

### Open systems

The horizontal-bore cylindrical type of scanner is still the commonest, but technology constantly changes and magnets are available with wider bores, which are less claustrophobic. More open scanners have been developed and units now exist that allow the patient to stand upright thus reducing the feeling of claustrophobia. In a conventional MR system operating at 1.5 T, because of its more closed design, it is less likely that radiological and anaesthetic staff would be exposed to significant static and time varying fields.

### Interventional procedures and intra-operative MR

Advances in technology mean MR image-guided surgery is now possible, providing the surgeon with dynamic high-resolution images during intricate stereotactic neurosurgery. Various MR systems have been configured for this application, including ‘doughnut’ shaped magnets permitting surgery with real-time concurrent imaging, and portable systems set up to allow easy and rapid interchange between scanning and surgery. All the hazards associated with diagnostic MR also apply to interventional procedures. There are additional risks from patient repositioning, contamination of the sterile field, and the proximity of ferromagnetic surgical instruments, including scalpels, to the magnetic field. Incorporating MR technology into the operating room provides new challenges [9].

## Occupational exposure

It is difficult to measure occupational exposure to the various electro magnetic fields in MR units routinely. Personal dosimeters have been developed but are not, as yet, widely available. Some studies have suggested that staff members can be exposed to higher than recommended levels of time-varying gradient fields [10, 11]. In 2004, the European Union adopted a directive restricting occupational exposure to electromagnetic fields, including those used in MR. Some of the exposure limits threatened to impact on the current use and future development of MR technology. Known adverse effects are adequately addressed in the international standard governing the manufacture of MR systems. Initially, unable to influence the regulatory agencies, the MR community began to lobby both the UK and European Parliaments. Implementation of the directive has been delayed until 30 April 2012 to allow a permanent solution to be found. However, the timescale is short given the political and scientific complexities of the issue. A range of possible outcomes is explored in a report for the Institute of Physics [12]. Each option has advantages and disadvantages, and a great deal of detailed discussion and negotiation will be needed over the next 2 years to ensure satisfactory resolution of the problem.

## Pacemakers and medical implants

The MHRA safety guidance [2] still specifies that pacemakers are an absolute contraindication to MR and it therefore remains the mantra of radiology departments that any individual with a pacemaker should not enter the MR unit. This is due to the concern that the magnet field strengths in excess of 0.5 mT (5 Gauss) could cause a fatal malfunction of the pacemaker. Sudden deaths have been reported in patients with pacemakers during or shortly after MR investigations [13, 14]. However, both pacemaker and MR technology are continually developing and there are times when MR is needed to provide valuable clinical information in patients with pacemakers. There have been a small number of cases when a patient with a non-compatible pacemaker has required MR imaging.

Approximately two million Europeans have implanted pacemakers, but these patients are strongly discouraged from receiving MRI scans. According to estimates, 50–75% of patients world-wide with implanted cardiac devices are expected to need a MR scan during the lifetime of their device [15]. Editorials in the American and European literature concluded that the risk:benefit ratio for patients with pacemakers undergoing MR has shifted towards safety, if guidelines are followed [16, 17]. Discussion in the correspondence sections has been generated [18]. In summary, the presence of a permanent pacemaker no longer represents a strict contra-indication to MR in carefully selected clinical circumstances provided that specific strategies are followed [19].

MR compatible pacemakers are now available and have been implanted in some patients. One pacemaker manufacturer has received a Conformité Européenne (CE) Mark for its second-generation MR safe pacing system. However, approval has not yet been forthcoming from the Food and Drug Administration in the USA [20].

### Programmable shunts

The pressure setting of programmable hydrocephalus shunts may be unintentionally changed by the magnetic field leading to over- or under-drainage of CSF. If these patients are to undergo an MR examination, a programmer and a trained clinician should be available to verify the correct setting and to reprogram the device, if required, immediately following the MR procedure. Advice must be given to the patient on how to recognise over- and under-drainage and whom to contact should these conditions develop [2].

### Neurostimulators

A wide variety of neurostimulators are now in use. Concerns about MR safety relate to the RF and gradient fields that may interfere with the operation of these devices or cause thermal injury. It is recommended that patients implanted with neurostimulators should not undergo MR. However, some manufacturers are suggesting that MR examinations of specific devices may be safe if strict guidelines relating to scanning parameters, in particular to RF exposure, are followed [2].

## Out-of-hours MR imaging

There are many indications for urgent MR imaging, but they can be grouped into two main areas: suspected spinal cord or cauda equina compression; and investigation of acute neurological conditions. Hospital trusts have faced litigation when treatment has been delayed due to lack of 24-h MR availability. Patients in intensive care units (ICUs) who require urgent MR will need to be accompanied by anaesthetic staff. Intensive care patients have additional sources of hazard including central lines and intracranial pressure transducers [21]. Screening checklists have been adapted for use in intensive care. It should be remembered that the ultimate operational responsibility for safety issues remains with an appropriately trained MR Authorised Person (usually the supervising radiographer) and the MR radiologist [2].

### Training

Training requirements for any staff entering the MR unit are detailed by the MHRA [2]. The responsibility for safety training lies with the MR Responsible Person who may be the clinical director, head of department, clinical scientist or MR superintendent radiographer. The unit’s MR Safety Advisor should provide technical advice. The wide range of staff from differing disciplines who need access to the MR environment have been designated into categories. Anaesthetists fall into MHRA category B; that is, they may be present with a patient in the MR controlled area during scanning. They should be aware of safety aspects related to the main static magnetic fields, RF fields, gradient magnetic fields and electrical safety of equipment. They must understand the significance of the MR controlled area and the inner MR controlled area. They should be familiar with emergency procedures arising from causes other than equipment failure and should be aware of the need to evacuate the patient from the inner controlled area in order to deal with emergency resuscitation. Training also includes an understanding of the projectile effect and the influence of the magnetic field upon medical implants, prostheses and personal effects.

Anaesthetists should understand the consequences of quenching of super-conducting magnets, and be aware of the recommendations on exposure to MR and the need for ear protection.

How can these training requirements be met? The potential for e-learning should be considered. The Royal College of Anaesthetists includes the physics of MRI in its basic science syllabus [22]. Trainees who have completed an e-learning module and attended an elective MR list would then be certified as suitable to accompany ICU patients for MR imaging. Regular reviews of training status as well as updates and refresher courses will be required. Hospitals will wish to apply local rules regarding consultant supervision of anaesthetic trainees in MR units.

## Contrast reactions

Gadolinium-based contrast agents are used in MR for demonstration of vascular structures or to improve contrast resolution of tissues. In comparison with other radiological contrast agents, Gd-CAs are relatively safe with a high therapeutic ratio and low incidence of anaphylaxis (approximately 1:100 000). The side-effects of Gd-CAs are generally mild and include headache, nausea and vomiting, local burning, skin wheals (2%), itching, sweating, facial swelling and thrombophlebitis [23]. More severe reactions have occurred and radiology staff should be familiar with guidelines related to the management of suspected anaphylaxis [24].

## Nephrogenic systemic fibrosis

There has been recent attention to reports that patients with renal failure are at risk of developing a rare, potentially life-threatening condition with Gd-CAs called nephrogenic systemic fibrosis or nephrogenic fibrosing dermopathy (NSF/NFD). The glomerular filtration rate (GFR) should be estimated in all patients with kidney disease to identify those at risk of developing NSF/NSD. If the GFR is estimated at less than 30 ml.min^−1^ per 1.73 m^−2^, the risk of Gd-CAs should be balanced against benefit, and a minimal dose of Gd-CAs only administered if an unenhanced scan proves insufficient [25]. The Gd-CA should not be administered again for at least 7 days. Current evidence suggests that contrast agents may be classified as high-risk, e.g. gadopentelic acid, medium-risk, e.g. gadobenic acid, and low-risk e.g. gadoteridol [26]. The use of high-risk agents is contra-indicated in neonates and during the peri-operative period in patients undergoing liver transplantation. The Gd-Cas are not recommended in pregnancy unless absolutely necessary.

## Conclusions

While some safety terminology has altered, the basic recommendations for provision of anaesthetic services in MR units have remained the same since first published in 2002 [1]. Anaesthetists who are involved with 3-T systems, open scanners or interventional and intra-operative procedures should remain acquainted with the constantly changing recommendations relating to occupational exposure. They should take all practical steps to minimise the risk from exposure. MR examinations of patients with pacemakers are no longer absolutely contra-indicated but may be carried out under strictly controlled conditions in exceptional cases. Increased requirements for MR imaging in intensive care and postoperative patients have increased the need for repeated training. The employment of e-learning modules may facilitate such training. There has been an increase in the number of allergic reactions to Gd-CAs and it is recognised that patients with renal failure are at risk of developing NSF.
